# A Moving Magnetic Grid-Type Long-Range Linear Absolute Displacement Sensor

**DOI:** 10.3390/s23020700

**Published:** 2023-01-07

**Authors:** Zhen Zhang, Lei Wang, Bingzhang Cao, Haoze Zhang, Jiawei Liu

**Affiliations:** School of Instrumentation Science and Engineering, Harbin Institute of Technology, Harbin 150001, China

**Keywords:** moving magnetic grid, permanent magnet array, hall sensor array, absolute displacement sensor

## Abstract

In order to overcome the shortcomings of the traditional magnetic absolute linear displacement sensors in which cables affect the flexibility and measurement range in linear motor transmission systems, this paper proposes a novel cable-free moving magnetic grid-type long-range absolute displacement sensor. The sensor consists of a magnetic grid and a signal acquisition board. The magnetic grid is a moving component that contains two rows of permanent magnet arrays, one for relative displacement measurement and the other for the displacement interval code. The signal acquisition board is a fixed component that uses *n* groups of two-row Hall sensor arrays for continuous absolute displacement measurement. The principle of the sensor using the 2D magnetic field signal for the relative displacement measurement is analyzed, and a measurement method based on Hall sensor arrays for coding and absolute displacement detection over *n* cycles is proposed. Finally, a sensor prototype is fabricated and the experiments are performed. The experimental results show that the measurement resolution of the sensor is 5 μm, and the measurement accuracy is ±14.8 μm within the measurement range of 0–98.3 mm. The proposed sensor can realize continuous absolute displacement measurement over multiple cycles without cable binding.

## 1. Introduction

Linear motor transmission systems are widely promoted and used as an emerging industrial application in modern intelligent and flexible production systems. The processing and transportation of a product by a system depend heavily on the precise positioning and feedback of the displacement measurement sensor [1]. The structure and performance of the displacement sensor directly affect the efficiency of the production system and the quality of the product [2,3]. The linear displacement sensor is the most basic measurement sensor in linear motor transmission systems [4], and the measurement type is mainly divided into absolute and incremental types [5,6]. Compared with incremental sensors, absolute displacement sensors have no accumulated error and do not need to find the initial position, and the absolute position can be read anytime [6]. Thus, they play an irreplaceable role in the displacement measurement system of industrial production [7,8].

The two main types of absolute displacement sensors most commonly used in linear motor transmission systems are optical grating and magnetic displacement sensors [9]. Optical grating sensors operate based on the interference and diffraction principle of light. They can realize high accuracy, high resolution, and fast response speed [10], and with the measurement error can reach ±0.275 μm over a 10 mm range and ±0.5 μm over a full 3040 mm range Magnetic displacement sensors operate based on the magneto-electric conversion principle using the properties of magnetic poles [11], and the measurement resolution can reach 0.01 μm over a range of 3040 mm [12]. Compared with optical grating displacement sensors, magnetic displacement sensors are widely used in industrial production [13] due to their simple structure, low cost, ability to work in harsh environments involving oil, dust, water, and temperature fluctuations, and better resistance to vibrations and shocks [14,15].

For magnetic absolute linear displacement sensors, Hao et al. [16] developed a sensor with an alternating pole structure, using six Hall sensors to acquire the magnetic signal in a three-phase manner and realize the absolute displacement measurement in a single cycle. Liu et al. [17] introduced a sensor with two rows of permanent magnet arrays, and a combination of coding and relative displacement measurement achieved absolute displacement measurement in a single-coded cycle. Ortner et al. [18] proposed an absolute long-range linear position system. The system uses four alternating permanent magnets as a magnetic source, and absolute encoding is performed by tilting the permanent magnet within the single measurement cycle. The sensor achieves the absolute displacement measurement by measuring the 3D magnetic field. Lin et al. [19] presented a magnetic array with two rows of different pole pitches for absolute displacement measurements. The absolute encoding is achieved by the offset between the magnetic poles. The sensor uses two MR sensors to measure the two rows of the magnetic arrays for absolute displacement measurements. Wu et al. [20] presented a sensor based on an orthogonal dual-traveling wave magnetic field. The sensor uses two field coils to generate the magnetic field, and two inductive coils induce the magnetic field to generate the electrical signals. The absolute position measurement is realized by comparing the phase difference of the electrical signals.

From the studies above, the traditional magnetic absolute linear displacement sensor usually consists of a magnetic grid and an encoded sensor [20,21]. The magnetic grid is the fixed component that provides the magnetic field information for the sensor. The encoded sensor with cable connection is the moving component. It is mounted on the motion unit for displacement calculation via the modulation of the magnetic field. In flexible linear motor transmission systems with large motion ranges and multi-motion unit collaboration, the cables of traditional magnetic sensors directly affect the travel range and performance of the motion units. The cable layout complicates the system structure and cannot be flexibly configured and used. Therefore, finding a long-range absolute linear displacement sensor without the influence of cables is an important problem that needs to be investigated.

In this paper, a novel moving magnetic grid-type absolute linear displacement sensor based on a Hall sensor array is constructed. The sensor consists of a magnetic grid with coded information segments and a signal acquisition board. The magnetic grid contains two rows of permanent magnetic arrays, one for recording the relative displacement during each magnetic field cycle and the other for encoding. The signal acquisition board comprises multiple groups of double-row Hall magnetic sensor arrays, which provide a displacement measurement reference for the sensor and can realize the detection and calculation of the absolute displacement across a long range and over multiple cycles.

## 2. Sensor Structure Design

**Figure 1:**
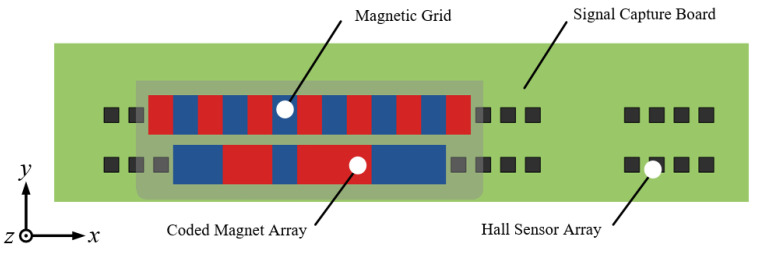
Schematic diagram of the sensor structure.

Figure 1 shows the structure of a moving magnetic grid-type absolute displacement sensor. The sensor consists of a magnetic grid and a signal acquisition board. The magnetic grid of the sensor is the moving component and comprises two parallel rows of permanent magnetic arrays. One row consists of multiple identically sized permanent magnets arranged in an alternating pole pattern to provide periodic magnetic field signals for relative displacement calculations. The other row consists of multiple permanent magnets of different lengths arranged in a binary coding pattern for the absolute coding of displacement. The signal acquisition board is a fixed component for the position detection and absolute displacement calculation of the sensor. The top side of the signal acquisition board is mounted with *n* groups of equally spaced Hall sensor arrays, each consisting of eight Hall sensors. Each Hall sensor array is divided into two rows, and each row comprises four Hall sensors. The signal acquisition board has a signal processing unit mounted on the bottom side for signal acquisition and processing of the *n* groups of Hall sensor arrays. The signal processing unit implements the absolute displacement calculation.

## 3. Principle of Displacement Measurement and Subdivision

**Figure 2:**
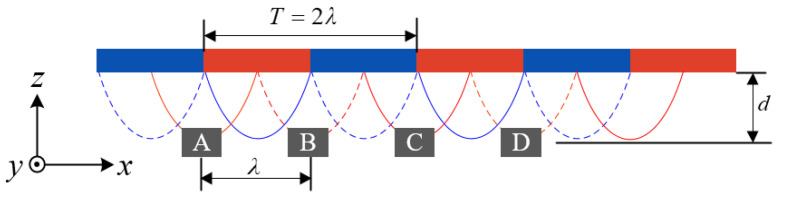
Sensor relative displacement measurement structure.

Figure 2 shows the relative displacement measurement structure of the sensor. The sensor uses a row of permanent magnets with alternating poles arranged in an N–S–N–S pattern to provide a periodic magnetic field signal. Four Hall sensors marked by “A”, “B”, “C”, and “D” are placed under the magnetic poles of the permanent magnet array for magnetic signal acquisition. The length of a single pole of the permanent magnet array is λ, the period is *T* = 2λ, and the spacing between Hall sensors is λ.

**Figure 3:**
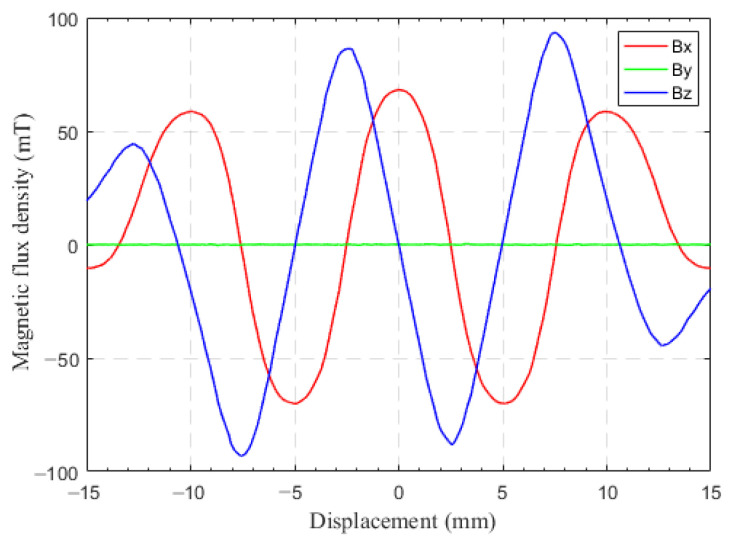
3D magnetic field of permanent magnet array detected by Hall sensor “A”.

The magnetic field of a permanent magnet array in space has three components: the *x*-axis, *y*-axis, and *z*-axis. The magnetic field of the permanent magnet array is simulated using ANSYS Electronics, and the 3D magnetic field signal of sensor “A” moving along the *x*-axis at a distance of 1.8 mm from the permanent magnet array is shown in Figure 3.

Assuming that the magnetic field generated by the permanent magnet array is an ideal periodic signal, without considering the edge effect and the influence of the harmonic magnetic field of the permanent magnetic array, the magnetic flux intensity detected by Hall sensor “A” at distance *d* from the permanent magnet array can be expressed as:

(1) $$B=\left[\begin{array}{c} B_{x}\\ B_{y}\\ B_{z} \end{array}\right]=\left[\begin{array}{c} B_{X}\cos(\frac{2\pi x_{s}}{T})\\ 0\\ -B_{Z}\sin(\frac{2\pi x_{s}}{T}) \end{array}\right]$$

where:

$B_{X}$ is the magnetic flux density amplitude on the *x*-axis;

$B_{Z}$ is the magnetic flux density amplitude on the *z*-axis;

$x_{s}$ is the relative displacement moving along the *x*-axis.

According to Equation (1), considering the effect of mechanical error and zero offset, the magnetic field signal of the Hall sensors “A”, “B”, “C”, and “D” along the *x*-axis and *z*-axis is:

(2) $$\left\{\begin{array}{c} B_{Ax}=B_{Cx}=\left(1+\alpha \right)B_{X}\cos\left(\frac{2\pi x_{s}}{T}\right)+\Delta \delta \\ B_{Az}=B_{Cz}=\left(1+\beta \right)B_{Z}\sin\left(\frac{2\pi x_{s}}{T}\right)+\Delta \varepsilon \\ B_{Bx}=B_{Dx}=-\left(1-\alpha \right)B_{X}\cos\left(\frac{2\pi x_{s}}{T}\right)+\Delta \delta \\ B_{Bz}=B_{Dz}=-\left(1-\beta \right)B_{Z}\sin\left(\frac{2\pi x_{s}}{T}\right)+\Delta \varepsilon \end{array}\right.$$

where:

α and β are the variation factor of swing;

Δδ and Δε is the zero drift.

The sensor array uses a differential structure to eliminate sensor errors and zero drift, where “A” and “B” are differential and “C” and “D” are differential. The Hall sensor output signal is corrected by the following equation:

(3) $$\left\{\begin{array}{c} B_{ABx}=\left(B_{Ax}-B_{Bx}\right)/2\\ B_{ABz}=\left(B_{Az}-B_{Bz}\right)/2\\ B_{CDx}=\left(B_{Cx}-B_{Dx}\right)/2\\ B_{CDz}=\left(B_{Cz}-B_{Dz}\right)/2 \end{array}\right.$$

When the sensor moves linearly along the *x*-axis, the magnetic field related to the direction of displacement motion is mainly the magnetic field components along the two directions of the *x*-axis and *z*-axis. Therefore, the displacement *x_s_* of linear motion along the *x*-axis obtained through a 2D magnetic field can be expressed as:

(4) $$x_{s}=\frac{T}{2\pi }\arctan\left(\frac{B_{z}}{B_{x}}\right)$$

**Figure 4:**
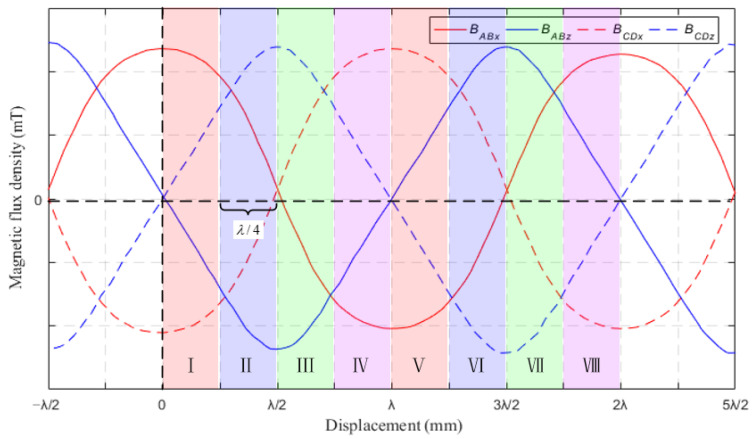
Schematic diagram of sensor displacement subdivision.

As shown in Figure 3, the magnetic flux density signals along the *x*-axis and *z*-axis of Hall sensors are orthogonal, and the phase differs by π/2. Therefore, a period *T* can be subdivided into eight intervals by using the Hall sensor to measure the 2D magnetic flux density signal of the magnetic array along the *x*-axis and *z*-axis. The range of displacement of each interval segment is λ/4. Figure 4 shows the sensor displacement subdivision, and the displacement *x_s_* for each interval can be calculated using Equation (4).

## 4. Principle of Sensor Multi-Cycle Displacement Coding

The relative displacement measurement is an incremental measurement of displacement. In the case of multi-cycle continuous displacement measurements, the position calculation is performed by counting and accumulating. When the sensor is powered off or started up, the displacement data used for counting accumulation will be lost, and the initial position cannot be determined independently. Therefore, a group of coded permanent magnet arrays is added to the magnetic grid. The absolute position measurement is achieved by independently encoding the subdivided relative displacement interval through the coded array.

**Figure 5:**
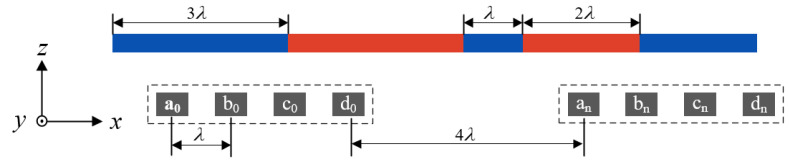
Coded permanent magnet array measurement structure.

Figure 5 shows the coded permanent magnet array measurement structure. The coded permanent magnet array consists of permanent magnets of different lengths arranged in an N–S–N–S pattern, and the entire length of the permanent magnet array is 11λ. The sensor uses *n* groups of sensor arrays arranged at a spacing distance of 4λ, and each group of sensor arrays used for coding contains four Hall sensors with a spacing of λ. The coding cycle of a single group coding array is $L_{s}=7\lambda$, where 3λ is the distance of a single coding array and 4λ is the intergroup spacing of the coding array. The Hall sensor array detects the pole’s magnetic field along the *z*-axis, and the magnetic signal is compared with the set threshold $T_{h}$ to determine the binary “0” and “1”, thus realizing the binary code.

The sensor binary coding judgment formula is:

(5) $$a_{n},b_{n},c_{n},d_{n}=\left\{\begin{array}{l} 0,B_{z}>T_{h}\\ 1,B_{z}\leq T_{h} \end{array}\right.$$

**Figure 6:**
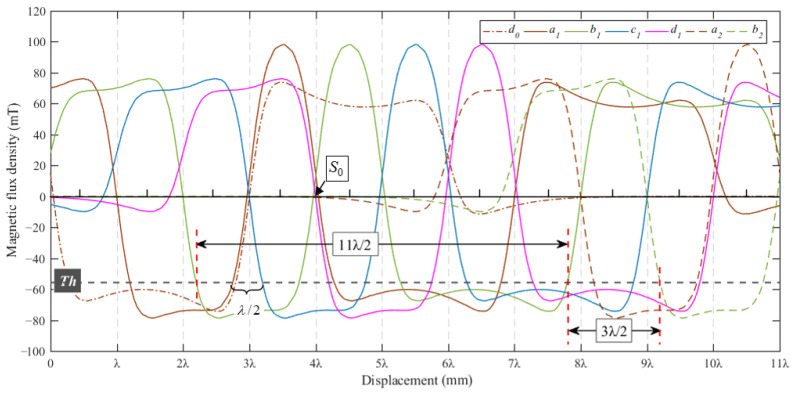
The waveform of the magnetic flux density of the coded Hall array.

Figure 6 shows the magnetic field waveforms of the adjacent three groups of Hall sensors (*d*_0_), (*a*_1_,*b*_1_,*c*_1_,*d*_1_), and (*a*_2_,*b*_2_) within the displacement interval 11λ of the magnetic grid shift. Setting a suitable threshold *T_h_* for coding allows a group of Hall sensor arrays to realize the binary coding of 11 consecutive displacement intervals. The measurement range of each displacement interval is λ/2, so the effective absolute displacement measurement range of a group of Hall sensor arrays is 11λ/2. As shown in Figure 6, the remaining 3λ/2 displacement within a coding cycle of 7λ requires the use of the adjacent group of Hall sensors to complete the encoding.

The displacement interval coding within a single cycle is shown in Table 1. In the process of the binary coding of displacement using Equation (5), the same coding displacement interval will appear, as is shown in column 3 of Table 1. Therefore, an interval code $e_{n}$ is added to ensure the uniqueness of the binary code. The interval code $e_{n}$ judgment formula is:

(6) $$e_{n}=\left\{\begin{array}{l} \begin{array}{c} 0,B_{d_{n-1}z}>Th\\ 1,B_{d_{n-1}z}\leq Th \end{array},index=1,8\\ \begin{array}{c} 0,B_{a_{n+1}z}\approx 0\\ 1,B_{a_{n+1}z}>S_{Th} \end{array},index=3,10\\ \begin{array}{c} 0,B_{a_{n+1}z}>Th\\ 1,B_{a_{n+1}z}\leq Th \end{array},index=12,13\\ 0,index=others \end{array}\right.$$

**Figure 7:**
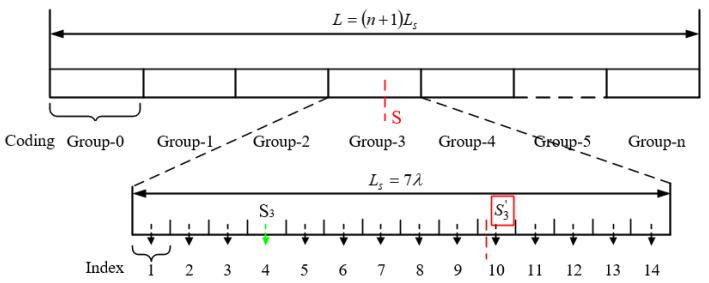
Schematic diagram of the absolute displacement measurement of the sensor.

In Equation (6), $S_{Th}$ is the judgment threshold and $S_{Th}>>0$. For continuous absolute displacement measurement over *n* coding cycles, the signal acquisition board provides the position measurement reference. When the magnetic grid array is in the displacement measurement region of the *n*-th group of Hall sensors arrays, its absolute displacement concerning the reference zero can be obtained from the absolute displacement in one coding cycle plus the displacement offset of the coding group. The absolute displacement measurement of the sensor is shown in Figure 7.

The calculation formula is as follows:

(7) $$S_{n}^{\prime }=S_{n}+k\lambda /2-2\lambda$$

(8) $$S=7\lambda n+S_{n}^{\prime },n\geq 0$$

where:

*S_n_* is the zero position of the *n*-th coding group;

$S_{n}^{\prime }$ is the absolute displacement within the *n*-th encoding group;

*n* is the coding group number;

*S* is the absolute displacement.

## 5. Experimental and Analysis Results

**Figure 8:**
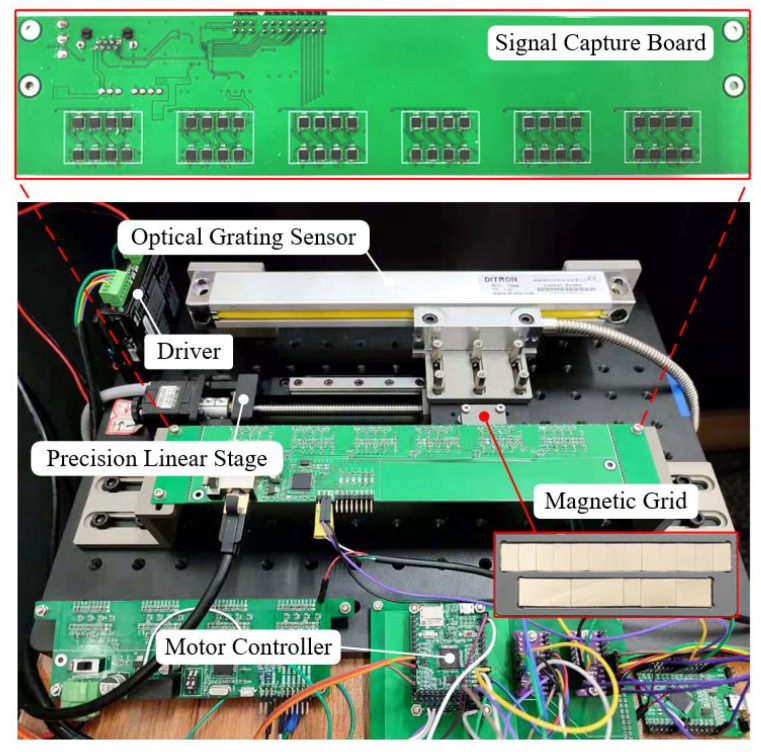
Experimental test platform.

Figure 8 shows the experimental platform and the sensor prototype. The experimental platform consists of an optical plate, a precision linear stage, an optical grating sensor, and a sensor prototype. The parameters of the experimental equipment are shown in Table 2. The material of the permanent magnet is NdFeB35, the length of the permanent magnet λ = 5 mm, and the thickness *h* = 0.8 mm. The signal acquisition board of the sensor prototype is made via a printed circuit board (PCB) multi-layer process, and multiple groups of Hall sensor arrays and signal processing circuits are integrated directly into the board. The board is mounted with six groups of Hall sensor arrays, and the distance between Hall sensors is λ = 5 mm. The coding measurement cycle of a single group of Hall sensor arrays *Ls* = 35 mm, so the total effective range of the absolute displacement measurement of the signal acquisition board is 210 mm. The Hall sensor uses the ALS31300EEJASR for magnetic signal acquisition with integrated internal ADC to provide a 12-bit digital output proportional to the magnetic field in each direction of the *x*, *y*, and *z*-axes. The vertical clearance between the magnetic grid and the signal acquisition board is *d* = 1.8 mm.

The readhead of the optical grating sensor and the magnetic grid of the sensor prototype are fixed on both sides of the slide block of the linear sliding stage and move together with the linear sliding stage. The signal acquisition board of the sensor prototype is fixed on the optical plate. During the experiment, incremental displacement data from the optical grating sensor and absolute displacement data from the sensor prototype are collected simultaneously and sent to a computer for comparison.

**Figure 9:**
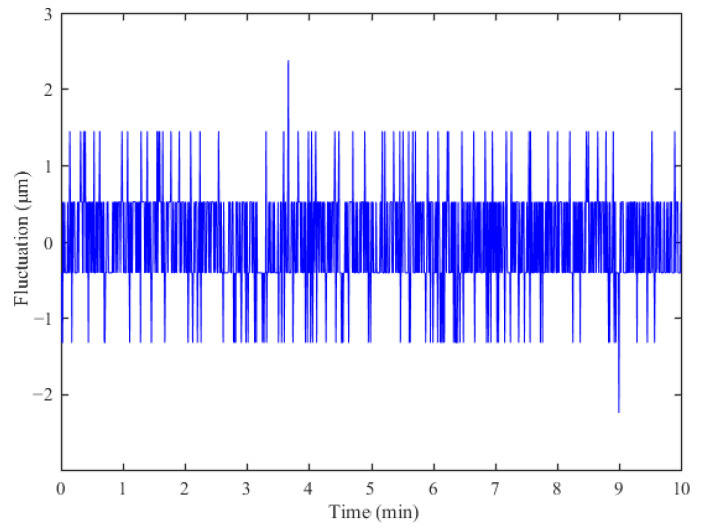
Measurement stability.

The linear slider stage is moved to a fixed position and records the absolute displacement data output from the sensor prototype. The measurement stability of the sensor is obtained by subtracting the displacement data from the average of the measurement result over the entire measurement time. Figure 9 shows the measurement stability result of the sensor prototype. The displacement fluctuation of the sensor was ±2.4 μm over 10 min. Therefore, the sensor has good measurement stability.

**Figure 10:**
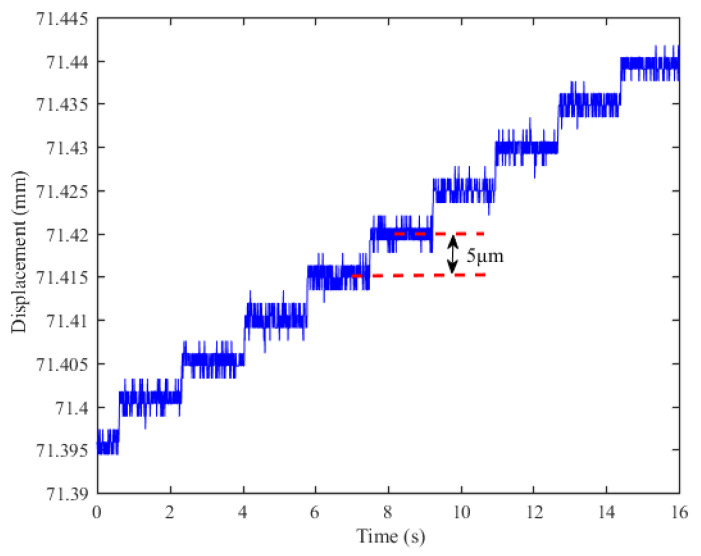
Resolution test results.

Figure 10 shows the results of the sensor resolution test. The displacement signal output from the sensor prototype is recorded by controlling the linear slide stage to move linearly in steps of 5 μm over a range of motion of 60 μm. The 5 μm step can be identified from the figure, so the resolution of the sensor is 5 μm. The resolution of the sensor prototype is mainly influenced by the resolution of the ADC inside the Hall sensor. Therefore, a better resolution can be achieved by using Hall sensors integrated with a higher ADC resolution.

**Figure 11:**
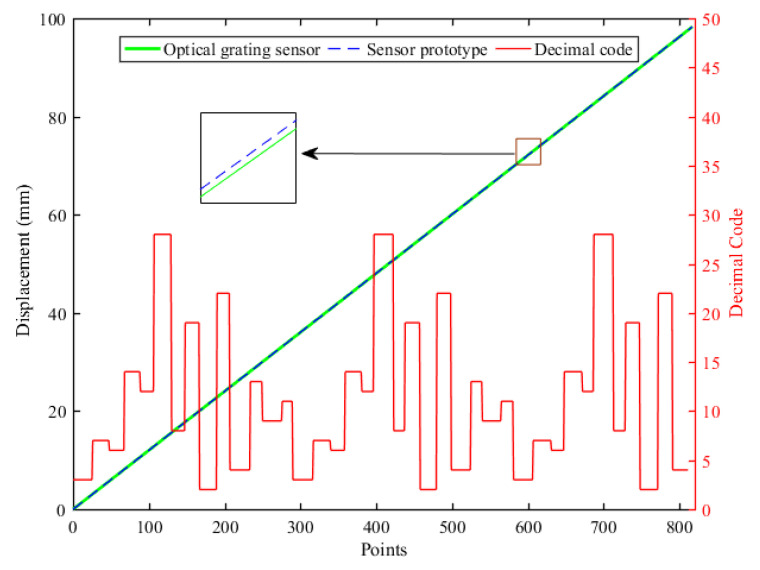
Sensor displacement measurement results and coding results.

**Figure 12:**
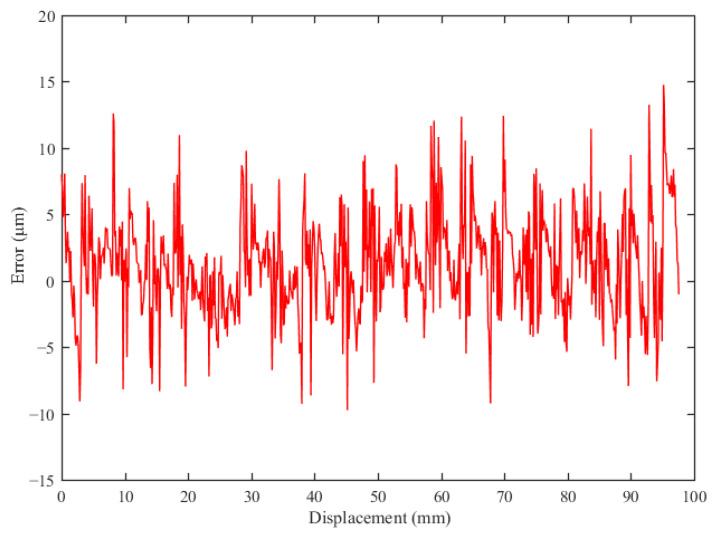
Sensor displacement measurement error.

Limited by the linear slider stage range and the structure of the whole experimental platform, the entire range of the sensor test is 98.3 mm. Over a motion range of 0–98 mm, 820 data points were plotted to evaluate the measurement performance of the sensor. The sensor displacement measurement results and the coding results are shown in Figure 11. As shown in Figure 11, the displacement measurement interval of the decimal code is not uniform. The measurement interval for a single code is approximately equal to λ/2, mainly due to manufacturing error in the permanent magnet and Hall sensor installation error. Figure 12 shows the error of the sensor prototype over the entire measurement range. The absolute displacement measurement error of the sensor is ±14.8 μm. The measurement error of the sensor prototype is a systematic component, mainly originating from the manufacturing tolerance of the permanent magnets and installation error of the Hall sensors. The irregularities in the size of the permanent magnet cause the phase and amplitude differences of sine and cosine in different periods. The position error of the Hall sensors installation causes deviation in the phase of the measured input signal.

**Figure 13:**
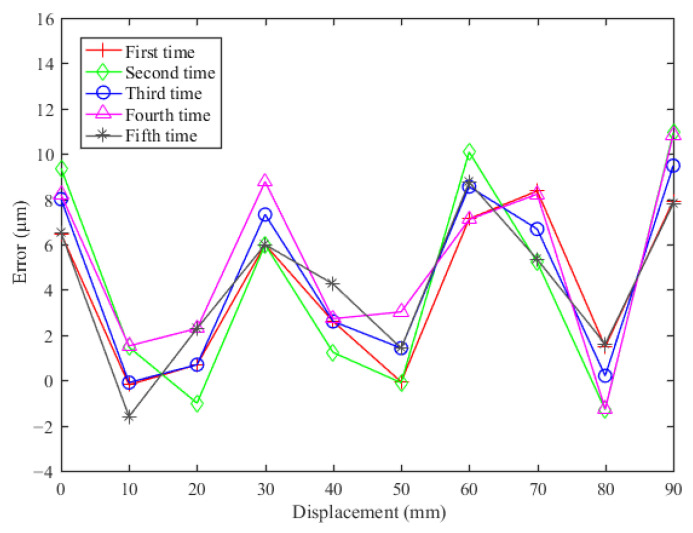
Sensor repeatability measurement result.

The repeatability experiment with the sensor was performed with a measuring step of 10 mm, a mechanical cycle with a measuring range of 0–98.3 mm, and five measurement repetitions. Figure 13 shows the repeatability measurement result of the sensor prototype. The standard deviation of the results was 1.5 μm.

## 6. Discussion

Table 3 shows the detailed parameter comparison with other magnetic absolute linear sensors and lists three types of magnetic absolute displacement with different working principles. Compared to the magnetic sensors in the table, the magnetic grid of the sensor prototype is the moving component, and the signal acquisition board mounted with the Hall sensor arrays is the fixed component, so the sensor is not constrained by cables. The measuring accuracy and resolution of the sensor prototype are low compared with the commercial magnetic sensor. However, it is only a prototype of the sensor, and some future measures will be taken to improve the performance of the sensor. The intrinsic error is caused by manufacturing tolerances and irregularity of Hall sensors and permanent magnets. These errors can be compensated with an error mapping technique to improve the sensor’s accuracy [22]. The resolution of the sensor can be improved by using Hall sensors integrated with a higher resolution ADC.

## 7. Conclusions

This paper proposed a novel moving magnetic grid-type absolute displacement sensor based on Hall sensor arrays. The sensor has no cable connection constraints and can achieve absolute displacement measurement over a long range. First, the structure of the sensor is presented. Then, the relative displacement measurement principle of the sensor is analyzed, and the method based on the Hall sensor arrays for coding and absolute displacement measurement over *n* cycles is established. Finally, the sensor prototype is manufactured, and the experimental platform is built for the performance test of the sensor. As a result, the sensor prototype is free of cable binding and achieves continuous absolute displacement measurement over multiple cycles. Compared with the traditional structure of the magnetic absolute displacement sensor, the sensor proposed in this paper has no cable interference, which improves the flexibility of the magnetic absolute displacement sensor. The characteristic of the sensor demonstrated excellent prospects for further applications to measure absolute linear displacement in flexible linear motor transmission systems with large motion ranges.

## Figures and Tables

**Table 1:** Absolute displacement code table.

Index	Interval Code	Binary Code ^1^	Decimal ^2^	Zero Position
0	0	0000	0	-
1	1	0011	19	S_n_ − 3λ/2
2	0	0010	2	S_n_ − λ
3	1	0110	22	S_n_ − λ/2
4	0	0100	4	S_n_
5	0	1101	13	S_n_ + λ/2
6	0	1001	9	S_n_ + λ
7	0	1011	11	S_n_ + 3λ/2
8	0	0011	3	S_n_ + 2λ
9	0	0111	7	S_n_ + 5λ/2
10	0	0110	6	S_n_ + 3λ
11	0	1110	14	S_n_ + 7λ/2
12	0	1100	12	S_n_ + 4λ
13	1	1100	28	S_n_ + 9λ/2
14	0	1000	8	S_n_ + 5λ

^1^ The binary code is in the order of $d_{n}c_{n}b_{n}a_{n}$. ^2^ The decimal is calculated in the order of $e_{n}d_{n}c_{n}b_{n}a_{n}$ binary code.

**Table 2:** Experimental equipment parameters.

Equipment	Model	Parameter 1	Value	Parameter 2	Value
Precision linear stage	LA100-60	Range	0–100 mm	Resolution	0.5 μm
Optical grating sensor	DC11-150MM	Range	0–150 mm	Resolution	1 μm
Hall Sensor	ALS31300EEJASR-1000	Range	±1000 G	Sensitivity	2 LSB/G
Stepper motor driver	SR2-PLUS	Subdivision	25,000 step/rev		

**Table 3:** Comparison with different types of magnetic absolute linear sensors.

	Type 1	Type 2	Type 3	Type 4	Type 5
Sensor	LA11	SR87	Sensor in [17]	Sensor in [20]	Sensor Prototype
Working principle	-	Magnetic Resistive	Hall effect	Magnetic Induction	Hall effect
Status	Commercial	Commercial	Laboratory	Laboratory	Laboratory
Producer	RLS	Magnescale	-	-	-
Moving component	Encoded sensor	Encoded sensor	Hall sensors	Inductive coil	Magnetic grid
Fixed component	Magnetic grid	Magnetic grid	Magnetic grid	Field coil	Hall sensor arrays
Accuracy	±10 μm	±3 μm	-	±15 μm	±14.8 μm
Resolution	0.244 μm	0.01 μm	0.75 μm	-	5 μm
Cable influence	Yes	Yes	Yes	Yes	No

## Data Availability

The data presented in this study are available on request from the corresponding author.
